# A clinical prediction model based on interpretable machine learning algorithms for prolonged hospital stay in acute ischemic stroke patients: a real-world study

**DOI:** 10.3389/fendo.2023.1165178

**Published:** 2023-11-22

**Authors:** Kai Wang, Qianmei Jiang, Murong Gao, Xiu’e Wei, Chan Xu, Chengliang Yin, Haiyan Liu, Renjun Gu, Haosheng Wang, Wenle Li, Liangqun Rong

**Affiliations:** ^1^ Department of Neurology, The Second Affiliated Hospital of Xuzhou Medical University, Xuzhou, Jiangsu, China; ^2^ Key Laboratory of Neurological Diseases, The Second Affiliated Hospital of Xuzhou Medical University, Xuzhou, Jiangsu, China; ^3^ Department of General Practice, Xindu District People’s Hospital of Chengdu, Chengdu, Sichuan, China; ^4^ Department of Rehabilitation, Beijing Rehabilitation Hospital Affiliated to Capital Medical University, Beijing, China; ^5^ Department of Dermatology, Xianyang Central Hospital, Xianyang, China; ^6^ Faculty of Medicine, Macau University of Science and Technology, Macau, Macao SAR, China; ^7^ School of Chinese Medicine and School of Integrated Chinese and Western Medicine, Nanjing University of Chinese Medicine, Nanjing, China; ^8^ State Key Laboratory of Pharmaceutical Biotechnology, Division of Sports Medicine and Adult Reconstructive Surgery, Department of Orthopedic Surgery, Nanjing Drum Tower Hospital, The Affiliated Hospital of Nanjing University Medical School, Nanjing, Jiangsu, China; ^9^ The State Key Laboratory of Molecular Vaccinology and Molecular Diagnostics and Center for Molecular Imaging and Translational Medicine, School of Public Health, Xiamen University, Xiamen, China

**Keywords:** prolonged hospital stay, stroke, machine learning, prediction model, SHAP (SHapley Additive exPlanations)

## Abstract

**Objective:**

Acute ischemic stroke (AIS) brings an increasingly heavier economic burden nowadays. Prolonged length of stay (LOS) is a vital factor in healthcare expenditures. The aim of this study was to predict prolonged LOS in AIS patients based on an interpretable machine learning algorithm.

**Methods:**

We enrolled AIS patients in our hospital from August 2017 to July 2019, and divided them into the “prolonged LOS” group and the “no prolonged LOS” group. Prolonged LOS was defined as hospitalization for more than 7 days. The least absolute shrinkage and selection operator (LASSO) regression was applied to reduce the dimensionality of the data. We compared the predictive capacity of extended LOS in eight different machine learning algorithms. SHapley Additive exPlanations (SHAP) values were used to interpret the outcome, and the most optimal model was assessed by discrimination, calibration, and clinical utility.

**Results:**

Prolonged LOS developed in 149 (22.0%) of the 677 eligible patients. In eight machine learning algorithms, prolonged LOS was best predicted by the Gaussian naive Bayes (GNB) model, which had a striking area under the curve (AUC) of 0.878 ± 0.007 in the training set and 0.857 ± 0.039 in the validation set. The variables sorted by the gap values showed that the strongest predictors were pneumonia, dysphagia, thrombectomy, and stroke severity. High net benefits were observed at 0%–76% threshold probabilities, while good agreement was found between the observed and predicted probabilities.

**Conclusions:**

The model using the GNB algorithm proved excellent for predicting prolonged LOS in AIS patients. This simple model of prolonged hospitalization could help adjust policies and better utilize resources.

## Introduction

With acute ischemic stroke (AIS) being the first leading cause of disability and the second leading cause of mortality worldwide, economic burden remains a prominent issue in clinical practice (1). Length of stay (LOS) is a vital factor of overwhelmed healthcare cost expenditures. Pellico-Lopez et al. (2 *)* found that 15.8% of the total cost of stroke cases depended on the cost of prolonged stay. Reducing unnecessary hospital stays is important to relieve insurance stress, especially under the policy of diagnosis-related groups (DRGs) payment. Therefore, it is essential that the risk model of prolonged LOS be analyzed to relieve economic burden and optimize the discharge plan for patients with AIS.

The average LOS following stroke onset varied according to time and country. In the United States, the LOS for stroke hospitalizations decreased from 2004 to 2018, according to the data survey of 8 million stroke patients (unadjusted: 6.3 days in 2004 vs. 5.6 days in 2018; adjusted: 7.6 days in 2004 vs. 5.4 days in 2018) (3). A *post-hoc* analysis (4) based on information from multiple sources in China found that the median and IQR of LOS for AIS was 10.0 (7.0–13.0) days. Hao et al. (5) found that malnutrition estimated by the CONUT score on admission could increase LOS in elderly AIS patients. Moreover, Neale et al. (6) found that stroke patients receiving an early supported discharge model of care spent fewer days in hospital and incurred less cost. In addition, the mode of treatment could also be related to the LOS after a stroke. Intravenous tissue plasminogen activator (IV-tPA) was associated with an increase in LOS in stroke patients treated with endovascular treatment within 4.5 h (7).

Only a few articles have currently established risk models for predicting the length of hospital stay in stroke patients. Koton et al. (8) evaluated the performance of the prolonged length of stay (PLOS) score in the cohort of stroke, and concluded that the PLOS score could be clinically useful in different healthcare systems. However, they only included patients from 2002 to 2007, and the treatments for stroke have developed dramatically in recent years. Nowadays, artificial intelligence is able to deduce from voluminous datasets and to incorporate nonlinear interactions among a large set of predictors (9–11). For machine learning predicting prolonged LOS in AIS, Kurtz et al. (12 *)* accurately predicted the LOS of patients admitted to the ICU with stroke through machine learning methods, but they did not include stroke-specific data, such as the National Institutes of Health Stroke Scale (NIHSS) score or neuroimaging findings. Yang et al. (13) found that the artificial neural network model achieved adequate discriminative power for predicting prolonged LOS after AIS and identified crucial factors associated with a prolonged hospital stay. However, they did not include pneumonia or another important onset symptom of stroke, which proved to be strong influencing factors of LOS in AIS patients.

As a result, we set out to gather extensive stroke-specific data and create a scientific risk model based on an interpretable machine learning algorithm to predict prolonged hospital LOS in AIS patients. This simple model of prolonged hospitalization could help adjust policies and better utilize resources.

## Methods

### Participant selection

**Figure 1:**
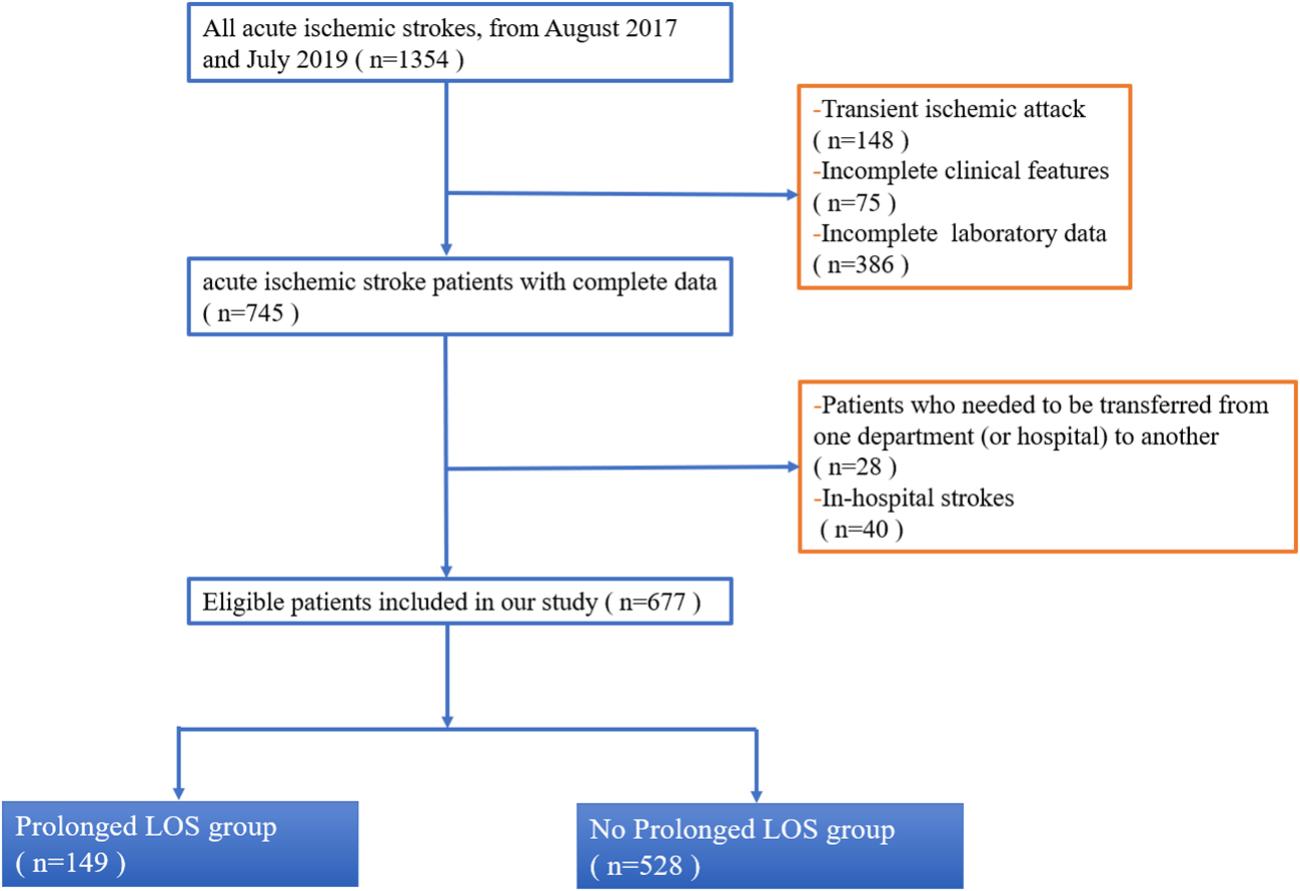
Flowchart of inclusion and exclusion of study patients. This flowchart indicated that our hospital managed about total 1,354 patients from August 2017 and July 2019, of which 745 (55%) AIS participants had complete data (**Figure 1**). Of these 745 patients, 68 patients were those who needed to be transferred from one department (or hospital) to another/those who had in-hospital strokes, leaving a final cohort of 677 patients. Abbreviation: LOS, length of stay.

This study continuously enrolled AIS patients who were admitted to the Department of Neurology at the Second Affiliated Hospital of Xuzhou Medical University between August 2017 and July 2019 (**Figure 1**). The inclusion criteria were as follows: (1) age ≥ 18 years; (2) a diagnosis of AIS (14, 15)and within 24 h of onset (16, 17). The exclusion criteria were as follows: (1) patients who needed to be transferred from one department (or hospital) to another; (2) patients who had in-hospital strokes; (3) patients who had transient ischemic attack; and (4) patients who were unable to extract complete data. This flowchart indicated that our hospital managed about a total of 1,354 patients from August 2017 and July 2019, of whom 745 (55%) AIS participants had complete data (**Figure 1**). Of these 745 patients, 68 patients were those who needed to be transferred from one department (or hospital) to another/those who had in-hospital strokes, leaving a final cohort of 677 patients. Retrospective review of medical health records for this study was approved by our Institutional Review Board. Owing to the retrospective nature of this study, written informed consent was waived (Number: 2020081603). Moreover, the Transparent Reporting of a Multivariable Prediction Model for Individual Prognosis or Diagnosis (TRIPOD) statements were followed for all data analysis and reporting (18).

### Data collection and definitions

The primary outcome was the prediction of a prolonged LOS for AIS patients, which was defined as more than 7 days of hospitalization. The LOS was measured from the admission day to the death or discharge day. This definition was similar to previous studies on LOS in stroke patients (8, 19, 20). The main clinical data included the following categories: baseline demographics, clinical features, and laboratory data. For baseline demographics, systolic blood pressure (SBP) and diastolic blood pressure (DBP) were tested on the right hand and extracted from the nursing record sheet on admission. For clinical features, stroke severity was divided into “mild” (NIHSS score < 8) and “moderate to severe” (NIHSS score ≥9), which was similar to previous clinical trials (21–23). Sato et al. (22 *)* found that the optimal cutoff score of the baseline NIHSS for the favorable outcome was 8 for patients with anterior circulation stroke (sensitivity, 80%; specificity, 82%). The pneumonia in our study referred to those with development of pneumonia within 72 h after hospitalization (24). We diagnosed pneumonia by the CDC criteria because it was the most commonly used (25). The dysphagia was defined as abnormal swallowing physiology of the upper aerodigestive tract and as detected from clinician testing including screening, clinical bedside, or instrumental tests (26). The thrombolysis, thrombectomy, antiplatelets, anticoagulation, statins, and proton pump inhibitors were also collected from medical records. Treatment methods for AIS were followed by the 2019 American Heart Association/American Stroke Association (AHA/ASA) guideline (27). For laboratory data, they were extracted from blood test results on admission.

### Machine learning algorithm and data analysis

Continuous data were presented as median and interquartile range (IQR), and the Mann–Whitney *U*-test was used for statistical comparison between two groups. Categorical data were described as proportions, and the chi-squared or Fisher’s exact test was used for comparison between two groups. The least absolute shrinkage and selection operator (LASSO) regression was applied to reduce the dimensionality of the data. In total, we utilized eight different machine learning algorithms, including the extreme gradient boosting (XGB) classifier, logistic regression, the light gradient boosting machine (LGBM) classifier, the AdaBoost classifier, Gaussian naive Bayes (GNB), complement naive Bayes (Complement NB), the multilayered perceptron (MLP) classifier, and the support vector (SVC) classifier. The hyperparameter settings for eight different machine learning algorithms used in our study are listed in **Supplementary Table 1**. For the XGB classifier, learning rate was set as 0.001, and the reg lambda was 0.01. Max depth and min child weight were set as 2. The area under the receiver operating characteristic (ROC) curve of the model was calculated by 10 bootstrapping resamples. For each bootstrap resample, the validation set (135 cases) accounted for 20% of the total sample, and the training set (542 cases) accounted for 80% of the total sample. After selecting the best model classifiers for this dataset, we exploited SHapley Additive exPlanations (SHAP) values to interpret the outcomes of the classifiers, which was a unified approach that connected cooperative game theory with local explanations to explain the output of any machine learning model. In addition, the decision curve analysis (DCA) was applied to present the net benefits at various threshold probabilities. A calibration plot was used to investigate the degree of agreement between two groups.

## Results

### Patient characteristics

**Figure 2:**
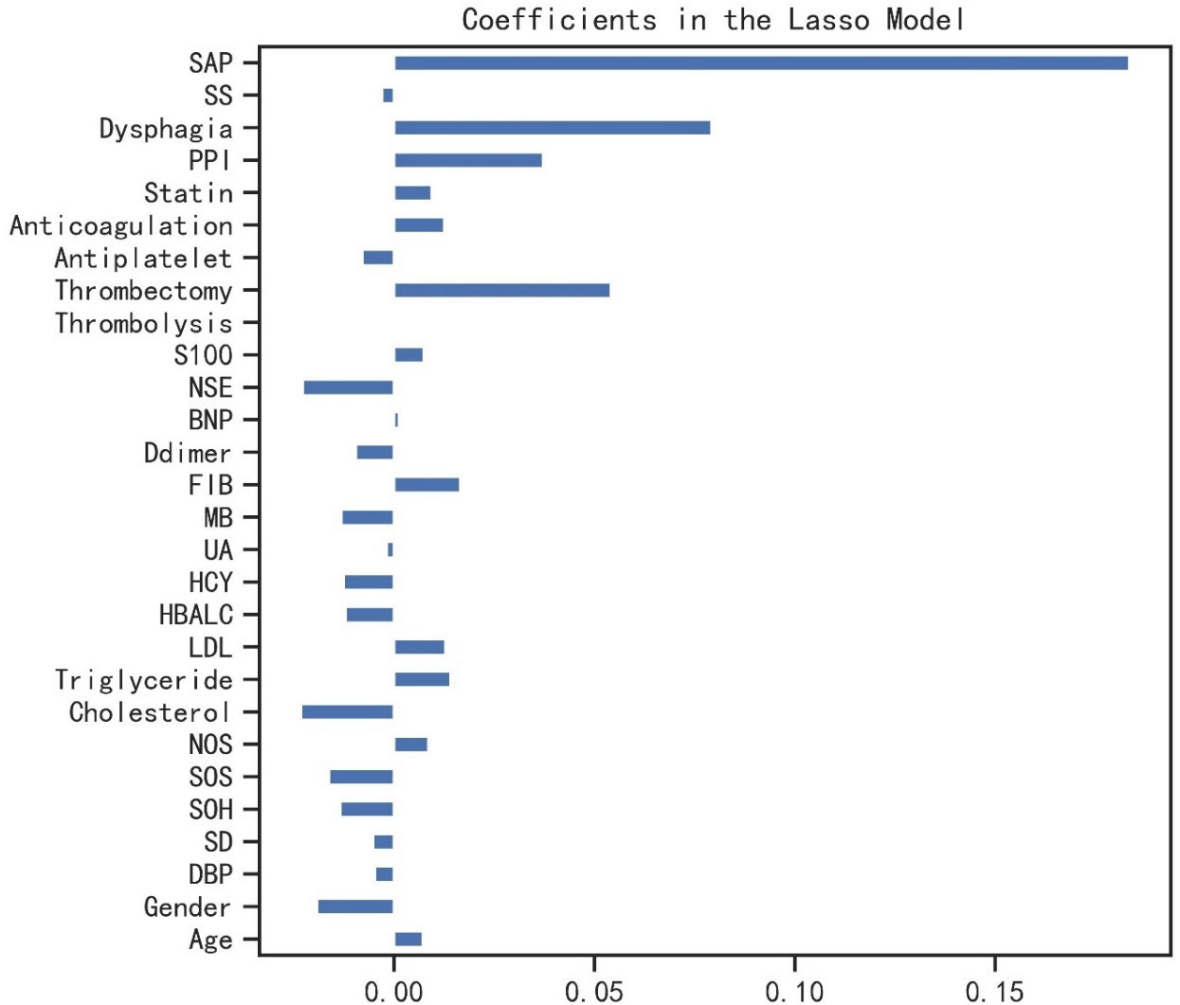
The coefficients of characteristics selected by LASSO regression. LASSO, least absolute shrinkage and selection operator; SAP, stroke-associated pneumonia; SS, stroke severity; PPI, proton pump inhibitor; NSE, neuron-specific enolase; BNP, b-type natriuretic peptide; FIB, fibrinogen; MB, myoglobin; UA, uric acid; HCY, homocysteine; HbA1c, glycosylated hemoglobin; LDL, low-density lipoprotein; NOS, number of stroke lesions; SOS, site of stroke lesion; SOH, **s**ide of hemisphere; SD, stroke distribution; DBP, diastolic blood pressure.

A total of 677 patients remained for evaluation of the machine learning algorithms to predict prolonged LOS in AIS patients, among whom prolonged LOS was detected in 22.0% (*n* = 149). The average of LOS in all 677 participants was 10.78 ± 4.69 days. The baseline and clinical characteristics between the two groups are compared in **Table 1**. Longer LOS was linked to elevated levels of brain natriuretic peptide (BNP), S100-β, and neuron-specific enolase (NSE). Moreover, the prolonged LOS group was more likely to suffer from dysphagia, pneumonia, and a moderate-to-severe stroke. As for treatment, the prolonged LOS group had more frequent use of thrombolysis, thrombectomy, anticoagulation, and proton pump inhibitors (PPIs). Then, least absolute shrinkage and selection operator (LASSO) regression was used to reduce the number of factors with an optimal λ of 0.002. The candidate characteristics were narrowed down to the following 28 features with nonzero coefficients: age, gender, diastolic blood pressure, anterior or posterior stroke, side of hemisphere, stroke lesion, single or multiple lesions, cholesterol, triglyceride, low-density lipoprotein (LDL), glycosylated hemoglobin (HbA1c), homocysteine (HCY), uric acid (UA), myoglobin (MB), and fibrinogen. The coefficients of characteristics selected by LASSO regression are illustrated in **Figure 2**.

**Table 1:** The baseline and clinical characteristics in prolonged LOS patients and no prolonged LOS patients.

Variables	Category	All patients (*n* = 677)	No prolonged LOS (*n* = 528)	Prolonged LOS (*n* = 149)	Statistical value	*p*
Demographics						
Age	NA	57 [45,68]	57 [45,68]	58 [47,68]	−1.210	0.226
Gender	Female	279 (41.21)	209 (39.58)	70 (46.98)	2.624	0.105
	Male	398 (58.79)	319 (60.42)	79 (53.02)		
SBP	NA	143 [132,156]	142 [132,155]	147 [135,159]	−2.302	0.021
DBP	NA	87 [74,97]	86 [73,96]	88 [76,98]	−1.251	0.211
Clinical features						
Stroke severity	Mild	385 (56.87)	339 (64.21)	46 (30.87)	52.637	<0.001
	Moderate to severe	292 (43.13)	189 (35.80)	103 (69.13)		
Dysphagia	No	525 (77.55)	465 (88.07)	60 (40.27)	152.495	<0.001
	Yes	152 (22.45)	63 (11.93)	89 (59.73)		
Stroke distribution	Anterior	270 (39.88)	208 (39.39)	62 (41.61)	0.737	0.692
	Posterior	252 (37.22)	201 (38.07)	51 (34.23)		
	Both	155 (22.90)	119 (22.54)	36 (24.16)		
Side of hemisphere	Left	283 (41.80)	223 (42.24)	60 (40.27)	0.462	0.794
	Right	270 (39.88)	207 (39.21)	63 (42.28)		
	Both	124 (18.32)	98 (18.56)	26 (17.45)		
Site of stroke lesion	Cortex	155 (22.90)	109 (20.64)	46 (30.87)	9.095	0.059
	Cortex-subcortex	155 (22.90)	125 (23.67)	30 (20.13)		
	Subcortex	186 (27.47)	151 (28.60)	35 (23.49)		
	Brainstem	104 (15.36)	86 (16.29)	18 (12.08)		
	Cerebellum	77 (11.37)	57 (10.80)	20 (13.42)		
Number of stroke lesions	Single	470 (69.42)	372 (70.46)	98 (65.77)	1.200	0.273
	Multiple	207 (30.58)	156 (29.55)	51 (34.23)		
Thrombolysis	No	473 (69.87)	385 (72.92)	88 (59.06)	10.598	0.001
	Yes	204 (30.13)	143 (27.08)	61 (40.94)		
Thrombectomy	No	644 (95.13)	525 (99.43)	119 (79.87)	95.943	<0.001
	Yes	33 (4.87)	3 (0.57)	30 (20.13)		
Antiplatelet	No	122 (18.02)	101 (19.13)	21 (14.09)	1.994	0.158
	Yes	555 (81.98)	427 (80.87)	128 (85.91)		
Anticoagulation	No	576 (85.08)	467 (88.45)	109 (73.15)	21.411	<0.001
	Yes	101 (14.92)	61 (11.55)	40 (26.85)		
Statin	No	103 (15.21)	84 (15.91)	19 (12.75)	0.898	0.343
	Yes	574 (84.79)	444 (84.09)	130 (87.25)		
PPI	No	535 (79.03)	462 (87.50)	73 (48.99)	103.954	<0.001
	Yes	142 (20.98)	66 (12.50)	76 (51.01)		
Pneumonia	No	512 (75.63)	473 (89.58)	39 (26.17)	253.486	<0.001
	Yes	165 (24.37)	55 (10.42)	110 (73.83)		
Laboratory data						
S-100β	NA	275 [224,290]	273 [221,288]	281 [237,297]	−3.057	0.002
NSE	NA	16.24 [12.69,18.60]	15.73 [12.61,18.43]	17.61 [14.05,18.92]	−3.196	0.001
BNP	NA	93 [73,162]	89 [73,158]	103 [77,168]	−2.213	0.027
D-dimer	NA	174 [133,221]	174 [134,219]	175 [132,224]	−0.239	0.812
FIB	NA	4.35 [3.96,4.75]	4.35 [3.95,4.71]	4.440 [4.04,4.79]	−1.488	0.137
CRP	NA	12.56 [7.72,17.63]	12.21 [7.63,17.19]	13.90 [8.11,19.06]	−1.956	0.050
MB	NA	97.66 [75.12,147.84]	98.77 [76.43,147.84]	94.85 [72.32,144.45]	0.864	0.388
UA	NA	349.80 [309.80,408.10]	353.20 [310.50,408.50]	343 [307.80,406.30]	0.595	0.552
HCY	NA	15.77 [12.74,19.37]	16.12 [12.54,19.31]	15.51 [13.04,20.01]	−0.525	0.600
HbA1c	NA	5.60 [5.30,5.90]	5.60 [5.40,5.90]	5.60 [5.30,6.00]	0.462	0.643
FBG	NA	5.28 [4.63,5.83]	5.28 [4.67,5.83]	5.22 [4.55,5.79]	1.099	0.272
LDL	NA	4.75 [4.33,4.94]	4.75 [4.31,4.95]	4.75 [4.37,4.90]	0.131	0.896
Triglyceride	NA	2.17 [1.93,2.37]	2.19 [1.93,2.37]	2.16 [1.93,2.37]	0.568	0.570
Cholesterol	NA	5.33 [4.43,6.15]	5.33 [4.40,6.11]	5.40 [4.54,6.25]	−1.075	0.283

LOS, length of stay; DBP, diastolic blood pressure; PPI, proton pump inhibitor; NSE, neuron-specific enolase; BNP, b-type natriuretic peptide; FIB, fibrinogen; CRP, C-reaction protein; MB, myoglobin; UA, uric acid; HCY, homocysteine; HbA1c, glycosylated hemoglobin; FBG, fasting blood glucose; LDL, low density lipoprotein; NA, not available.

### Development and validation of models

**Figure 3:**
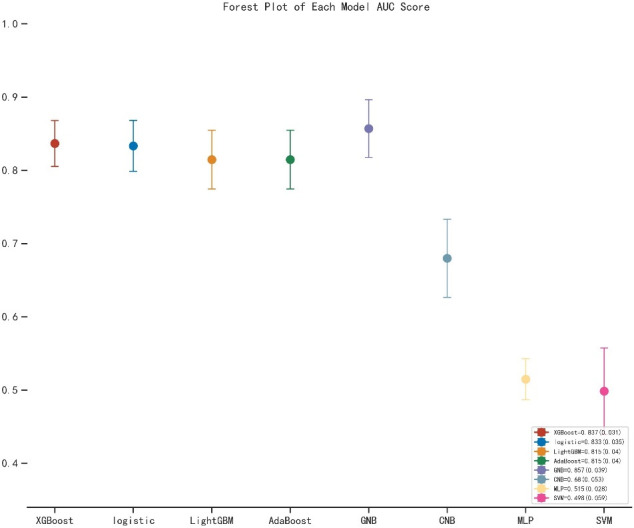
The forest plot of the each AUROC of eight models. AUROC, area under the receiver operating characteristic curve; XGB, extreme gradient boosting; LGBM, light gradient boosting machine; GNB, Gaussian naive Bayes; CNB, complement naive Bayes; MLP, multilayered perceptron; SVM, support vector machine.

**Figure 4:**
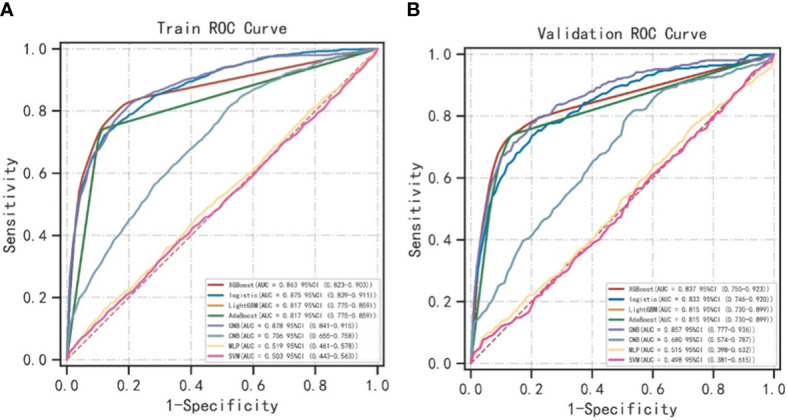
The comparison of AUROC between the GNB model and the other seven models. **(A)** The comparison of AUROC between the GNB model and the other seven models in the training sets. **(B)** The comparison of AUROC between the GNB model and the other seven models in the validation sets. ROC, area under the receiver operating characteristic curve; XGB, extreme gradient boosting; LGBM, light gradient boosting machine; GNB, Gaussian naive Bayes; CNB, complement naive Bayes; MLP, multilayered perceptron; SVM, support vector machine.

**Figure 5:**
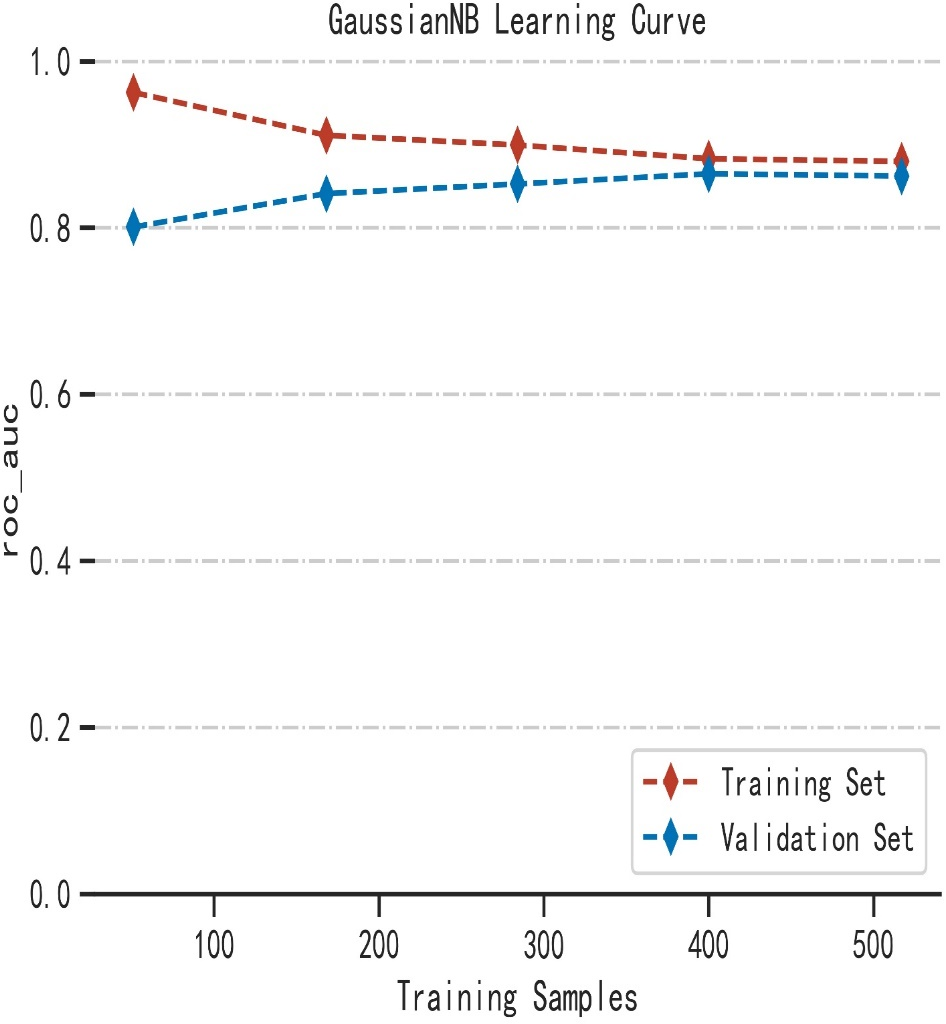
The learning curve of the GNB model.

As shown in **Table 2**, the GNB model with all characteristics had a striking AUROC of 0.878 ± 0.007 in the training set and 0.857 ± 0.039 in the validation set, while the other seven representative models had the highest AUROC of 0.875 ± 0.014 in the training set and 0.837 ± 0.031 in the validation set. For the GNB model, the sensitivities were 0.818 (training sets) and 0.804 (validation sets), while the specificities were 0.814 (training sets) and 0.816 (validation sets). The cross-reference between the full names and abbreviations in our manuscript is shown in **Supplementary Table 2**. The forest plot of each AUROC of eight models is depicted in **Figure 3**. **Figures 4A, B** present the comparison of AUROC between the GNB model and the other seven models, respectively, in the training and validation sets. The learning curve of the GNB model is displayed in **Figure 5**. Obviously, the GNB model significantly outperformed the other seven models in both the training and validation sets. Despite the narrow gap, De Long’s test showed that the difference between the GNB and XGB model remained significant (*p* = 0.04).

**Table 2:** The predictive capacity of eight different machine learning algorithms.

	Model	AUC (SD)	Accuracy (SD)	Sensitivity (SD)	Specificity (SD)	PPV (SD)	NPV (SD)	Kappa (SD)
	XGB	0.863 (0.011)	0.862 (0.009)	0.799 (0.027)	0.845 (0.033)	0.671 (0.045)	0.923 (0.008)	0.609 (0.021)
	logistic	0.875 (0.014)	0.837 (0.019)	0.752 (0.042)	0.863 (0.032)	0.608 (0.046)	0.924 (0.009)	0.561 (0.034)
	LGBM	0.817 (0.009)	0.782 (0.008)	0.739 (0.016)	0.895 (0.007)	NA	0.782 (0.008)	0.000 (0.000)
Train	AdaBoost	0.817 (0.009)	0.782 (0.008)	0.739 (0.016)	0.895 (0.007)	NA	0.782 (0.008)	0.000 (0.000)
set	GNB	0.878 (0.007)	0.813 (0.020)	0.818 (0.030)	0.814 (0.030)	0.551 (0.039)	0.939 (0.007)	0.533 (0.037)
	CNB	0.706 (0.028)	0.613 (0.075)	0.747 (0.145)	0.577 (0.133)	0.337 (0.036)	0.896 (0.031)	0.222 (0.046)
	MLP	0.519 (0.045)	0.626 (0.147)	0.401 (0.287)	0.690 (0.266)	0.314 (0.098)	0.809 (0.018)	0.072 (0.045)
	SVM	0.503 (0.033)	0.658 (0.112)	0.304 (0.227)	0.761 (0.207)	0.274 (0.051)	0.798 (0.019)	0.052 (0.030)
	XGB	0.837 (0.031)	0.862 (0.023)	0.759 (0.052)	0.877 (0.052)	0.682 (0.091)	0.917 (0.019)	0.606 (0.090)
	logistic	0.833 (0.035)	0.813 (0.034)	0.750 (0.080)	0.840 (0.102)	0.575 (0.105)	0.906 (0.022)	0.501 (0.095)
	LGBM	0.815 (0.040)	0.774 (0.031)	0.730 (0.073)	0.900 (0.028)	NA	0.774 (0.031)	0.000 (0.000)
Validation	AdaBoost	0.815 (0.040)	0.774 (0.031)	0.730 (0.073)	0.900 (0.028)	NA	0.774 (0.031)	0.000 (0.000)
set	GNB	0.857 (0.039)	0.791 (0.036)	0.804 (0.035)	0.816 (0.075)	0.527 (0.098)	0.926 (0.022)	0.487 (0.098)
	CNB	0.680 (0.053)	0.582 (0.073)	0.740 (0.173)	0.609 (0.181)	0.316 (0.047)	0.862 (0.052)	0.166 (0.037)
	MLP	0.515 (0.028)	0.599 (0.157)	0.463 (0.308)	0.680 (0.300)	0.274 (0.145)	0.787 (0.039)	0.039 (0.050)
	SVM	0.498 (0.059)	0.636 (0.142)	0.559 (0.338)	0.560 (0.330)	0.243 (0.122)	0.772 (0.040)	0.014 (0.064)

AUC, area under the curve; SD, standard deviation; PPV, positive predictive value; NPV, negative predictive value; XGB, extreme gradient boosting; LGBM, light gradient boosting machine; GNB, Gaussian naive bayes; CNB, complement naive Bayes; MLP, multilayered perceptron; SVM, support vector machine; NA, not available.

### SHAP values depending on variables

**Figure 6:**
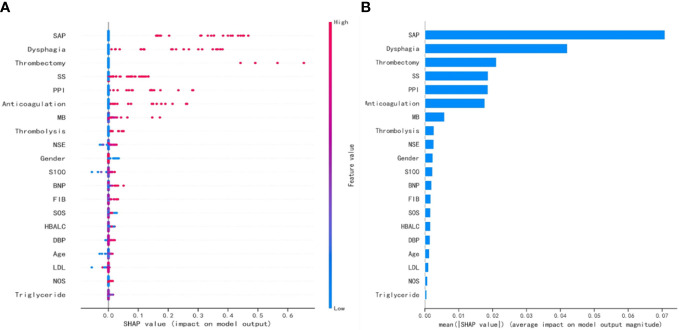
The SHAP values for the GNB model and the importance ranking of the variables. **(A)** The SHAP values for the GNB model. **(B)** The importance of the variables sorted by the gap values. SHAP, SHapley Additive exPlanations; GNB, Gaussian naive Bayes; SAP, stroke-associated pneumonia; SS, stroke severity; PPI, proton pump inhibitor; MB, myoglobin; NSE, neuron-specific enolase; BNP, b-type natriuretic peptide; FIB, fibrinogen; SOS, site of stroke lesion; HbA1c, glycosylated hemoglobin; DBP, diastolic blood pressure; LDL, low-density lipoprotein; NOS, number of stroke lesions.

**Figure 7:**
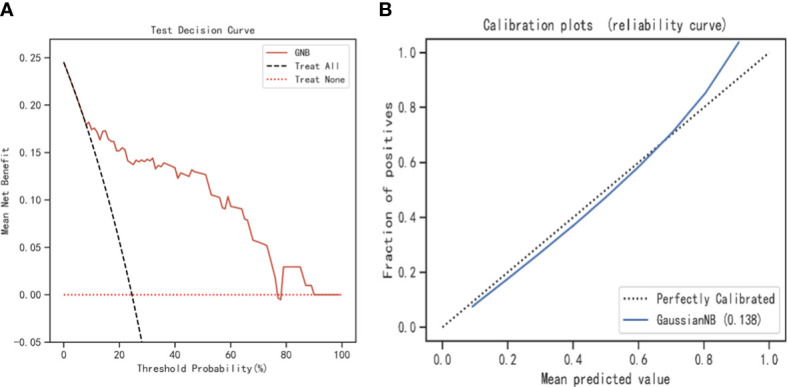
The decision curve analysis and calibration plot to illustrate the performance of the GNB model. **(A)** The decision curve analysis for the GNB model. **(B)** Calibration plot for the GNB model. GNB, Gaussian naive Bayes.

The SHAP values for the GNB model and the importance of the variables sorted by the gap values are shown in **Figures 6A, B**. Red bars indicated an increase in the probability of prolonged LOS, whereas blue bars demonstrated a decrease in the probability of prolonged LOS for AIS patients. As **Figure 6B** shows, pneumonia, dysphagia, thrombectomy, and stroke severity all substantially increased the probability of prolonged LOS. In addition, we performed a decision curve analysis (**Figure 7A**) and a calibration plot (**Figure 7B**) to illustrate the performance of the GNB model. High net benefits could be observed in 0%–76% threshold probabilities, while good agreement could be found between the observed and predicted probabilities of prolonged LOS.

## Discussion

This study generated a simple clinical risk model that can be used to determine patients at increased risk of prolonged LOS. Our risk model had a promising AUC of 0.878 and 0.857 in the training and validation sets, respectively. The main outcomes of the current study were that pneumonia, dysphagia, thrombectomy, and stroke severity were the strongest clinical parameters for prolonged LOS following AIS after recursive feature elimination. Moreover, the artificial intelligence algorithms developed by these parameters showed excellent model performance on discrimination, calibration, and decision curve analysis. The strengths of our clinical risk score included the use of simple demographic and common biochemical parameters, and we collected enough candidate variables to develop this model. To our knowledge, this is the first study to predict prolonged LOS for common AIS patients based on an interpretable machine learning algorithm. The difference from previous studies was that we developed an integrated machine learning model with high performance, which could help adjust the policies to better utilize resources, especially under the DRG payment policy and the increasingly serious aging problem in the global world.

Su et al. (28) included 129,444 patients with AIS and found that the inpatient cost was $1,020 ($742–$1,545) in China. In an attempt to decrease patients’ risk of prolonged LOS following AIS, previous retrospective studies have identified some factors. Many studies define prolonged LOS as more than 7 days (8, 19, 20). However, when it comes to patients with severe strokes or those admitted to an intensive care unit, some studies define it as more than 30 days (12, 29). Common factors affecting stroke hospitalization duration included quality of care, hospital-acquired infection, stroke severity and type, level of consciousness, history of heart failure and atrial fibrillation, and receiving reperfusion therapy (19, 29–33). Interestingly, during adolescence, low stress resilience, underweight, and higher systolic blood pressure were associated with longer hospital stays in AIS, with adjusted relative hazard ratios of 1.46, 1.41, and 1.01, respectively (34), whereas these prior studies did not show the weight of each parameter on the probability of prolonged LOS. An interpretable machine learning algorithm has the ability to analyze big datasets with high accuracy through automated analysis of non-linear relationships between numerous variables (35). Machine learning algorithms apply various statistical methods from past experience to select useful patterns in large and complex datasets, which involves extreme gradient boosting (XGB) classifier, GNB, SVC classifier, and so on (36). Raizada et al. (37) concluded the advantages and limitations of different algorithms and found that GNB produced results that were statistically robust and were replicates across two independent datasets. An additional advantage of GNB classifiers was that GNB produced an accuracy similar to more sophisticated classifiers but with a substantial gain in speed (38). Therefore, we selected the GNB model from eight different machine learning algorithms that showed excellent performance in predicting prolonged LOS in AIS patients.

In this study, pneumonia, dysphagia, thrombectomy, and stroke severity were the leading clinical parameters in our interpretable machine learning algorithm. Pneumonia is an early complication of stroke and usually leads to prolonged LOS. The prevalence of pneumonia in patients with dysphagia after stroke was reported to range from 7% to 33%, and the prevalence of dysphagia has been reported as between 28% and 65% (39, 40). Aspiration without a cough, known as “silent aspiration,” further increased the incidence of pneumonia to 54% (40). A systematic review of stroke-associated pneumonia reported that the overall incidence of pneumonia ranged from 0% to 23.6% (41), which was a little lower than the incidence in our study. In our study, the incidence of pneumonia in all participants is 24.37%. It may be because of the varied definitions and diagnosis criteria of stroke-associated pneumonia. The Centers for Disease Control and Prevention (CDC) criteria (25), the PISCES SAP diagnostic criteria (42), and the combination of the clinical symptoms and auxiliary examination results criteria were all used to diagnose stroke-associated pneumonia in previous studies (41). In our study, we diagnosed pneumonia by the CDC criteria because it was the most commonly used, using clinical (lung auscultation and percussion, presence of fever, and purulent tracheal secretion), microbiological (tracheal specimens and blood cultures), and chest radiography findings. For dysphagia, the incidence in all participants was 22.45%, while in the “prolonged LOS group”, it was 59.73%, and in the “no prolonged LOS group”, it was 11.93% (**Table 1**). The incidence of dysphagia varied greatly between studies (ranged from 20% to 80%), depending on the definition of dysphagia, which can range from failing a dysphagia screen, to prescribed diet modifications, to measures of physiology on an instrumented swallowing study (26, 41, 43). Ogawa et al. (40) found that patients who underwent a flexible endoscopic evaluation of swallowing and received optimal nutritional intervention were more likely to have a shorter hospital stay (*p* = 0.005). The complications of dysphagia include the consequences of modifications to dietary intake: compromised nutrition and hydration, prolonged LOS, and reduced quality of life. As a result, the optimal treatments and measures for dysphagia should be performed. Many studies have investigated a variety of interventions, including therapist-delivered, behavioral, acupuncture, and electrical or magnetic stimulation to treat dysphagia (39). As for stroke severity, it was the most consistent factor among the factors contributing to LOS in AIS patients, and those who received reperfusion therapy were more likely to have prolonged LOS, which was similar to the previous study (29). Patients with more severe strokes may require more intensive medical care, including medication treatment and rehabilitation. Thrombectomy is a procedure used to remove a blood clot from a blood vessel, and is typically used in the treatment of acute ischemic stroke. While thrombectomy can be effective in reducing the severity of stroke and improving patient outcomes, it is also a relatively invasive procedure that can carry some risks and complications. As a result, patients who undergo thrombectomy may require longer hospital stays than those who do not. In summary, both thrombectomy and stroke severity are independent risk factors for prolonged LOS following AIS.

Our study has several limitations. First, its retrospective study design and only including patients from one single tertiary central hospital may limit the generalizability of the machine learning algorithm in clinical practice. Second, owing to the availability of the data, we were not able to consider more detailed factors, such as specific steps of reperfusion therapy, infarction or penumbra volume, and the collateral circulation status. More valuable and dynamic predictors could improve the performance. Third, some special reasons that might affect hospitalization time, such as economic stress or medical disputes, were not analyzed. Fourth, the sample size and certain bias limited the predictive ability of the model. We just internally validated our interpretable machine learning algorithms by bootstrap resample and multi-center large-sample studies are warranted to verify this conclusion in the future.

## Conclusion

We developed a model for predicting the prolonged LOS for AIS patients using the GNB algorithm. This model included 20 potential clinical factors and performed well in terms of discrimination, calibration, and clinical utility, but it needs to be validated in larger multicenter cohorts. In this model, pneumonia, dysphagia, thrombectomy, and stroke severity might be strong predictors of prolonged LOS. We explained these main variables and analyzed the effects of their changing trends on prolonged LOS. Timely prevention and intervention for complications, as well as high quality standard of care, may be prospects worthy of clinicians’ promising efforts.

## Data availability statement

The raw data supporting the conclusions of this article will be made available by the authors, without undue reservation.

## Ethics statement

The studies involving human participants were reviewed and approved by Ethics Committee of the Second Affiliated Hospital of Xuzhou Medical University. The patients/participants provided their written informed consent to participate in this study.

## Author contributions

WL, LR, and HW completed the study design. KW and WL performed the study, and collected and analyzed the data. QJ and WL drafted the manuscript. LR, XW, KW, and HL provided the expert consultations and suggestions. MG, RG, CX, and CY conceived the study, participated in its design and coordination, and helped to embellish language. All authors contributed to the article and approved the submitted version.

## Supplementary material

Supplementary Table 1 The hyperparameter settings for eight different machine learning algorithms. Abbreviations: XGB, extreme gradient boosting; LGBM, light gradient boosting machine; GNB, Gaussian naive Bayes; CNB, complement naive Bayes; MLP, multilayered perceptron; SVM, support vector machine.

Supplementary Table 2 The cross-reference between the full names and abbreviations in our manuscript.
