# Supplementary material for: A clinical prediction model based on interpretable machine learning algorithms for prolonged hospital stay in acute ischemic stroke patients: a real-world study
Source: Front Endocrinol (Lausanne). 2023 Nov 22;14:1165178. doi: 10.3389/fendo.2023.1165178 (PMC10703471; doi:10.3389/fendo.2023.1165178)
Supplement: Supplementary Table 1 — The hyperparameter settings for eight different machine learning algorithms. Abbreviations: XGB, extreme gradient boosting; LGBM, light gradient boosting machine; GNB, Gaussian naive Bayes; CNB, complement naive Bayes; MLP, multilayered perceptron; SVM, support vector machine. [file DataSheet_1.docx]

**ONLINE SUPPLEMENT**

**A clinical prediction model based on interpretable machine learning algorithms for prolonged hospital stay in acute ischemic stroke patients: a real-world study**

**Supplementary Table 1.** The hyperparameter settings for eight different machine-learning algorithms.

**Supplementary Table 2.** The cross-reference between the full name and abbreviations in our manuscript.

**Supplementary Table 1.** The hyperparameter settings for eight different machine-learning algorithms.

| Model | Parameter settings |
| --- | --- |
| XGB classifier: | objective: binary logistic |
|  | Learning rate: 0.001 |
|  | Max depth: 2 |
|  | Min child weight: 2 |
|  | Reg lambda: 0.01 |
| Logistic regression | C: 1 |
|  | Max iter: 100 |
|  | Penalty: l2 |
|  | Tol: 0.0001 |
| LGBM classifier: | Boosting type: gbdt |
|  | Learning rate: 0.001 |
|  | Max depth: 1 |
|  | N estimators: 5 |
|  | Num leaves: 5 |
| AdaBoost classifier: | Learning rate: 0.001 |
|  | N estimators: 50 |
| GNB classifier: | Priors: None |
|  | Var smoothing: 1e-07 |
| CNB classifier: | Alpha (laplace/lidstone): 0 |
| MLP classifier: | Activation: logistic |
|  | Hidden layer sizes: (10, 10) |
|  | Max iter : 20 |
| SVM classifier: | C: 1.0 |
|  | Kernel: rbf |
|  | Tol: 0.001 |

Abbreviations: XGB, extreme gradient boosting; LGBM, light gradient boosting machine; GNB, Gaussian naive bayes; CNB, complement naive bayes; MLP, multilayered perceptron; SVM, support vector machine.

**Supplementary Table 2**. The cross-reference between the full names and abbreviations in our manuscript.

| **Abbreviation** | **Full name** |
| --- | --- |
| AIS | Acute ischemic stroke |
| AHA/ASA | American Heart Association/American Stroke Association |
| AUC | Area under the curve |
| BNP | Brain natriuretic peptide |
| CNB | Complement naive bayes |
| DBP | Diastolic blood pressure |
| DCA | Decision curve analysis |
| DRGs | Diagnosis-related groups |
| FBG | Fasting blood glucose |
| FIB | Fibrinogen |
| GNB | Gaussian naive bayes |
| HbA1c | Glycosylated hemoglobin |
| HCY | Homocysteine |
| IQR | Interquartile range |
| IV-tPA | Intravenous tissue plasminogen activator |
| LASSO | Least absolute shrinkage and selection operator |
| LDL | Low density lipoprotein |
| LGBM | Light gradient boosting machine |
| LOS | Length of stay |
| MB | Myoglobin |
| MLP | Multilayered perceptron |
| NA | Not available |
| NIHSS | National Institutes of Health Stroke Scale |
| NOS | Number of stroke lesions |
| NSE | Neuron-specific enolase |
| PLOS | Prolonged length of stay |
| PPI | Proton pump inhibitors |
| SAP | Stroke associated pneumonia |
| SBP | Systolic blood pressure |
| SD | Stroke distribution |
| SHAP | Shapley additive explanations |
| SOH | Side of hemisphere |
| SOS | Site of stroke lesion |
| SS | Stroke severity |
| SVC | Support vector |
| TRIPOD | Transparent Reporting of a Multivariable Prediction Model for Individual Prognosis or Diagnosis |
| UA | Uric acid |
| XGB | Extreme gradient boosting |
